# Supplementary material for: KEVS: enhancing segmentation of visceral adipose tissue in pre-cystectomy CT with Gaussian kernel density estimation
Source: Int J Comput Assist Radiol Surg. 2025 May 9;20(7):1335–44. doi: 10.1007/s11548-025-03380-7 (PMC12226616; doi:10.1007/s11548-025-03380-7)
Supplement: Supplementary file 1 — (pdf 121 KB) [file 11548_2025_3380_MOESM1_ESM.pdf]

Supplementary materials (“Online Resource 1”) for IPCAI Paper ID 76: *KEVS: Enhancing Segmentation of Visceral Adipose Tissue in Pre-Cystectomy CT with Gaussian Kernel Density Estimation*

Online Resource Table 1: A table displaying the F1 and ROC AUC for five predictive models after five-fold cross validation and Bayesian hyper-parameter optimisation for three different binary predictive tasks; the incidence of a pulmonary, renal, or infectious adverse event three days post surgery. The results compare the models trained on just patient characteristic data (CHAR), CHAR with additional *KEVS* predicted VAT data (CHAR+VAR), and CHAR data with full *KEVS* frailty prediction analysis (CHAR+FULL), including statistics for muscle, SAT, and bone in addition to VAT.

		CHAR		*CHAR+VAT*		*CHAR+FULL*	
**Complication**	**Classifier**	ROC AUC	F1	ROC AUC	F1	ROC AUC	F1
Pulmonary	Logistic Regression	0.6594 ± 0.1034	0.7604 ± 0.0347	0.6594 ± 0.1034	0.7604 ± 0.0347	$\mathbf{0.6935 \pm 0.0578}$	$\mathbf{0.7638 \pm 0.0247}$
	Random forest	0.6234 ± 0.1045	0.7569 ± 0.0329	0.6130 ± 0.0867	0.7623 ± 0.0202	$\mathbf{0.7273 \pm 0.0881}$	$\mathbf{0.7695 \pm 0.0133}$
	Support vector classifier	0.6416 ± 0.1073	0.7586 ± 0.0000	0.6571 ± 0.0724	$\mathbf{0.7659 \pm 0.0175}$	$\mathbf{0.6987 \pm 0.0905}$	0.7586 ± 0.0000
	Decision Tree	0.6377 ± 0.0848	$\mathbf{0.7552 \pm 0.0827}$	0.6351 ± 0.0812	0.6586 ± 0.1016	$\mathbf{0.6558 \pm 0.0334}$	0.7310 ± 0.0463
	XGBoost	0.6156 ± 0.0825	0.7586 ± 0.0000	0.5390 ± 0.1330	$\mathbf{0.7811 \pm 0.0275}$	$\mathbf{0.7039 \pm 0.0859}$	0.7775 ± 0.0714
Renal	Logistic Regression	0.4610 ± 0.0296	$\mathbf{0.4651 \pm 0.0910}$	$\mathbf{0.5400 \pm 0.0288}$	0.4551 ± 0.0455	0.4577 ± 0.1056	0.4180 ± 0.1311
	Random Forest	0.6249 ± 0.1256	$\mathbf{0.4065 \pm 0.1183}$	0.5282 ± 0.1413	0.4056 ± 0.2212	$\mathbf{0.6445 \pm 0.0798}$	0.3785 ± 0.2426
	Support vector classifier	0.5420 ± 0.1231	$\mathbf{0.4721 \pm 0.1012}$	$\mathbf{0.5944 \pm 0.0524}$	0.3802 ± 0.1080	0.5933 ± 0.0444	0.3607 ± 0.0760
	Decision Tree	0.6196 ± 0.0934	0.4849 ± 0.1535	$\mathbf{0.6255 \pm 1046}$	$\mathbf{0.5385 \pm 0.0644}$	0.5317 ± 0.0296	0.3621 ± 0.1049
	XGBoost	0.4523 ± 0.1404	0.2044 ± 0.1791	$\mathbf{0.5872 \pm 0.0970}$	0.3038 ± 0.1955	0.5749 ± 0.1436	$\mathbf{0.4964 \pm 0.1738}$
Infectious	Logistic Regression	0.6326 ± 0.0898	0.7921 ± 0.0526	$\mathbf{0.6338 \pm 0.0736}$	$\mathbf{0.7943 \pm 0.0070}$	0.6159 ± 0.0906	0.7841 ± 0.0384
	Random forest	$\mathbf{0.7016 \pm 0.1209}$	0.8155 ± 0.0190	0.6444 ± 0.0627	0.8155 ± 0.0190	0.5856 ± 0.0767	$\mathbf{0.8218 \pm 0.0259}$
	Support vector classifier	$\mathbf{0.6782 \pm 0.0508}$	0.8155 ± 0.0190	0.6280 ± 0.0751	0.8155 ± 0.0190	0.5856 ± 0.0767	0.8133 ± 0.0166
	Decision Tree	0.6521 ± 0.0935	$\mathbf{0.8407 \pm 0.0542}$	$\mathbf{0.6606 \pm 0.1523}$	0.7902 ± 0.0908	0.5133 ± 0.1523	0.7780 ± 0.0866
	XGBoost	0.6265 ± 0.1457	0.8155 ± 0.0190	$\mathbf{0.6295 \pm 0.2029}$	0.8155 ± 0.0190	0.5046 ± 0.0598	0.8155 ± 0.0190

Online Resource Table 2: A table displaying the different categories included in the "CHAR" data used to train the machine learning classifiers, with median and inter-quartile range (IQR) for numerical and categorical data, and absolute number and percentage for binary data. To produce "CHAR+VAT", VAT area at each lumbar vertebrae, and VAT volume between the vertical bounds of the lumbar vertebrae were included in addition to "CHAR" data. For "CHAR+FULL", muscle and SAT area and volume were taken in the same way, and also average and standard deviation of HU intensity values for each of muscle and the lumbar vertebrae.

**Numerical**	**Median (IQR)**
Age (years)	71.0 (58.5-76.0)
Weight (kg)	77.0 (68.4 - 86.4)
Height(m)	1.69 (1.64 - 1.75)
BMI (kg/m^2)	26.6 (24.0-30.4)
Number cigarettes smoked daily	15 (0-20)
Years smoked	20 (0-35)
Creatinine (µmol/L)	84.5 (71-103)
Haemoglobin (g/L)	129 (117 - 141)
Sodium (mmol/L)	140 (141 - 137.25)
Potassium (mmol/L)	4.4 (4.7 - 6.4)
Urea (mmol/L)	7.5 (6.4 - 7.5)
White blood cells (x 10^9/L)	8.1 (6.8 - 9.3)
**Categorical**	**Median (IQR)**
Operation severity	3 (2-3)
Operative access	2 (2-2)
Malignancy	2 (2-4)
American Society of Anaesthesiology grade (ASA)	3 (2-3)
Ethnicity Key	5.5 (1 - 17)
**Binary**	**Number (%)**
Sex (Female)	28 (31%)
Myocardial infarction	47 (52.2%)
Angina	2 (2.2%)
Coronary stent	2 (2.2%)
Hypertension	39 (43.3%)
Chronic Heart Failure	26 (28.9%)
Previous coronary artery bypass graft (CABG)	2 (2.2%)
COPD	4 (4.4%)
Asthma	7 (7.8%)
Smoking	14 (15.5%)
Athritis	11 (12.2%)
Beta blockers	10 (11.1%)
ACE Inhibitor	14 (15.6%)
Statins	34 (37.8%)
Diabetes	21(23.3%)
Peripheral vascular disease	2 (2.2%)
High risk surgery	80 (88.9%)
ECG Ischaemia rest	3 (3.3%)
ECG Ischaemia exercise	29 (32.3%)
Previous cerebrovascular accident/transient ischaemic attack	5 (5.6%)
